# Relationships between sickle cell trait, malaria, and educational outcomes in Tanzania

**DOI:** 10.1186/s12879-017-2644-x

**Published:** 2017-08-15

**Authors:** Kevin Croke, Deus S. Ishengoma, Filbert Francis, Julie Makani, Mathias L. Kamugisha, John Lusingu, Martha Lemnge, Horacio Larreguy, Günther Fink, Bruno P. Mmbando

**Affiliations:** 1 Harvard T. H. Chan School of Public Health, Boston, USA; 2 National Institute of Medical Research, Tanga Research Centre, Tanga, Tanzania; 3 Department of Haematology and Blood Transfusion, Muhimbili University of Health and Allied Sciences, Dar es Salaam, Tanzania; 4 Department of Government, Harvard University, Cambridge, USA

## Abstract

**Background:**

Sickle Cell Trait (SCT) has been shown to be protective against malaria. A growing literature suggests that malaria exposure can reduce educational attainment. This study assessed the relationship and interactions between malaria, SCT and educational attainment in north-eastern Tanzania.

**Methods:**

Seven hundred sixty seven children were selected from a list of individuals screened for SCT. Febrile illness and malaria incidence were monitored from January 2006 to December 2013 by community health workers. Education outcomes were extracted from the Korogwe Health and Demographic Surveillance system in 2015. The primary independent variables were malaria and SCT. The association between SCT and the number of fever and malaria episodes from 2006 to 2013 was analyzed. Main outcomes of interest were school enrolment and educational attainment in 2015.

**Results:**

SCT was not associated with school enrolment (adjusted OR 1.42, 95% CI [0.593,3.412]) or highest grade attained (adjusted grade difference 0.0597, 95% CI [−0.567, 0.686]). SCT was associated with a 29% reduction in malaria incidence (adjusted IRR 0.71, 95% CI [0.526, 0.959]) but not with fever incidence (adjusted IRR 0.905, 95% CI [0.709-1.154]). In subgroup analysis of individuals with SCT, malaria exposure was associated with reduced school enrollment (adjusted OR 0.431, 95% CI [0.212, 0.877]).

**Conclusions:**

SCT appears to reduce incidence of malaria. Overall, children with SCT do not appear to attend more years of school; however children who get malaria despite SCT appear to have lower levels of enrolment in education than their peers.

## Background

While a relatively large literature has highlighted the negative consequences of cerebral malaria on children’s cognitive development, evidence on the impact of repeated exposure to uncomplicated malaria infections remains scarce [1]. A growing literature has documented the importance of human genetic variations in the exposure to, and transmission of, malaria. Genes with protective traits against malaria have been shown to occur with increased frequencies in malaria-endemic regions. Among the genetic variations which offer protection against malaria are those that determine red blood cell (RBC) haemoglobin disorders in general, and those that cause thalassaemia and sickle cell disease (SCD) in particular. SCD is a classic example of a balanced polymorphism: although the heterozygous state of the sickle cell gene (HbAS) confers protection against malaria, the homozygous state of the sickle gene (SS) is associated with increased morbidity and mortality [2–5]. Subjects with one allele (HbAS – the sickle cell *trait,* hereafter referred to as *SCT*) are generally perceived to not suffer immediate negative health consequences, but to benefit from protection from malaria infection and mortality. There is also suggestive evidence that these protective effects translate into cumulative health benefits such as reduced rates of stunting [5]. Both SCD and SCT are very common in sub-Saharan Africa, and have been demonstrated to occur with high frequency in areas with high malaria transmission.

There is a large literature suggesting that malaria explains an important component of the lagging development performance of sub-Saharan Africa [6]. Using micro data, several recent papers [7–12] show long run benefits to cohorts exposed to malaria control or eradication programs early in life with respect to educational attainment, cognition, employment, and/or earnings. However, these studies largely rely on ecological designs; for example several compare educational outcomes for individuals born in more versus less malarial areas prior to national eradication campaigns. As such they may be subject to confounding biases. Research which examines educational outcomes as a function of individual, rather than geographic, variation in malaria exposure is needed.

The genetic variations generated by SCD provide an opportunity to identify the effect of malaria exposure in childhood on educational attainment. Using the technique of Mendelian randomization, the key assumption is that a specific genotype (in this case HbAS) is linked to a health-related characteristic (protection from malaria), but is unrelated to other confounding variables or to the outcome of interest [13]. If this assumption is valid, then individuals with SCT will have reduced exposure to malaria but will otherwise be comparable to individuals without sickle cell trait. This property of SCT has been previously used, in a Mendelian randomization framework, to study the relationship between malaria and stunting [14], but has not to our knowledge been used to study the relationship between malaria and educational attainment. If it is true that exposure to non-severe malaria reduces children’s cognitive development and ability to learn, children with the SCT living in highly endemic malaria areas should therefore display improved educational outcomes in the long run. This study therefore utilized genetic and epidemiological data to assess the effects of exposure to malaria and SCT on children’s educational attainment in an area that was until recently holo/hyper-endemic to malaria (Korogwe district in north-eastern Tanzania.)

## Methods

### Study area

The data used in this study was collected in Korogwe district in north-eastern Tanzania. Korogwe district is topographically stratified into lowland and highland areas with altitude ranging from 300 to 1200 m above sea level, and a population of 310,346. The district is characterized by varying malaria transmission with areas in the lowlands having high transmission, where *Plasmodium falciparum* is the dominant malaria species [15, 16]. Tanzania’s National Institute for Medical Research (NIMR) has been running a Health and Demographic Surveillance System (HDSS) in 14 villages with a population of more than 28,000 people, since January 2006 [17]. Out of 14 villages, six have been participating in surveillance of febrile episodes using community health workers known as community owned resource persons (CORPs) [18]. Three of these villages (Kwashemshi, Mkokola and Mng’aza) are in the lowland areas with traditionally high malaria transmission, and three villages (Kwamasimba, Kwamhanya and Magundi) are in the highland areas with low malaria transmission. Two of these villages (Kwamasimba and Mkokola) started the passive case detection (PCD) of febrile episodes in 2003 [19], while in the remaining four villages the surveillance was introduced in January 2006. Over 30,000 febrile illnesses have been recorded from the six villages since January 2006. Data from the HDSS shows that by 2013, the number of households in the six villages in which PCD of fever was operational was 3221, with a total population of 14,049 people.

Seven hundred sixty seven individuals aged 0–19 years were selected from a malariometric cross sectional survey conducted between May 2006 and May 2007 for genotyping of different malaria-associated polymorphisms including SCD. Genotyping was done by the MalariaGEN genomic epidemiology network. Educational attainment information was obtained for 704 (91.7%) of these individuals through the HDSS system up to May 2015. Genotype data was collected specifically for research purposes, malaria and fever diagnosis data was collected as part of the implementation of the passive case detection system of febrile illness, and education and other socioeconomic status indicators were collected through the routine procedures of the Korogwe Health and Demographic Surveillance System. Permission was obtained to use the data for this study.

#### Outcome variables

The primary outcome variables analyzed were a continuous measure of educational attainment, defined as highest grade of schooling attained, and a binary measure school enrolment, both measured as of 2015. Secondary outcome variables were febrile illness and malaria over the period 2006–2013.

#### Independent variables

The primary independent variables of interest were the presence of the SCT and malaria. Given the possibility that educational outcomes and malaria morbidity could be correlated with location and with socioeconomic status, control variables that proxy for socioeconomic status (such as access to electricity and piped water, asset ownership, and quality of housing) as well as village and ethnic group indicator variables are included in adjusted models as control variables.

### Empirical analysis

Multivariable Poisson regression models were used to analyze the associations between SCT and the number of febrile illnesses as well as the number of malaria episodes. For education, the association between SCT and grade attainment was estimated controlling for each age-year category. As an alternative empirical model, the probability that a child was still in school when last surveyed in 2015 was analyzed using standard logistic models.

For all models, both unadjusted estimates and adjusted estimates controlling for child age and sex, village, ethnic group, and a range of additional socioeconomic characteristics are shown. Robust standard errors are clustered at the village level. All empirical analysis was conducted using the Stata SE 13 software package.

## Results

Table 1 shows descriptive statistics for the sample. Mean age of individuals in the sample was 15.9 years as of May 2015. Approximately half of the individuals (56%) were female. In the SCT group, 36% of sampled individuals lived in a household that owns a bicycle and 16% owned a phone, 38.7% had piped water, 2.7% had electricity in their homes. For the non-SCT sample, 29.6% owned bicycles and 8% owned phones, while 0.5% had electricity and 27.9% had piped water in their homes. The adjusted models presented in Tables 2, 3 and 4 use all of the variables in Table 1 as controls.

**Table 1:** Characteristics of the sample

	Group			
	HbAA (*N* = 623)		HbAS (*N* = 81)	
Age of child (years), Mean (SD)	15.9	(4.0)	15.5	(3.7)
Child is female, *n* (%)	350	(56.1)	49	(60.5)
Village: Kwamasimba,*n* (%)	171	(27.4)	19	(23.5)
Village: Kwamhanya, *n* (%)	43	(6.9)	3	(3.7)
Village: Magundi, *n* (%)	77	(12.4)	8	(9.9)
Village: Mkokola, *n* (%)	162	(26.0)	33	(40.7)
Village: Mng’aza, *n* (%)	49	(0.1)	9	(0.1)
Village: Kwashemshi,*n* (%)	121	(0.2)	9	(0.1)
Ethnicity: Sambaa, *n* (%)	339	(54.4)	40	(49.4)
Ethnicity: Zigua, *n* (%)	53	(8.5)	9	(11.1)
Household has bike, *n* (%)	173	(29.6)	28	(36.0)
Household has radio, *n* (%)	415	(71.1)	55	(73.3)
Household has phone, *n* (%)	46	(8.0)	12	(16.0)
Household has brick walls, *n* (%)	264	(45.5)	23	(32.0)
Household has electricity, *n* (%)	3	(0.5)	2	(2.7)
Household has piped water, *n* (%)	162	(27.9)	29	(38.7)
Household has toilet, *n* (%)	523	(89.7)	66	(88.0)
Head is employed (*n*%)	21	(3.6)	8	(10.7)
Land area cultivated (acres), Mean (SD)	2.3	(1.6)	2.5	(2.1)

Socioeconomic status (SES) variables available for *N* = 584 HbAA and *N* = 75 HbAS

**Table 2:** Sickle cell trait and incidence of fever and malaria

	Number of fever episodes		Number of malaria episodes	
	Unadjusted Incidence Rate Ratio (IRR)	Adjusted Incidence Rate Ratio (IRR)	Unadjusted Incidence Rate Ratio (IRR)	Adjusted Incidence Rate Ratio (IRR)
Sickle cell trait	0.897 (0.647–1.244)	0.905 (0.709–1.154)	0.861 (0.610–1.214)	0.710** (0.526–0.959)
Number of observations	704	654	704	654

Notes: Adjusted models include all covariates listed in Table 1; dummy variables for each year of age are also included. Robust standard errors clustered at village level. ** *p* < 0.05

**Table 3:** Sickle cell trait and educational outcomes

	Child is currently in school		Grade attained conditional on age	
	Unadjusted Odds Ratio (OR)	Adjusted Odds Ratio (OR)	Unadjusted grade difference	Adjusted grade difference
Sickle cell trait	1.339 (0.676–2.654)	1.423 (0.593–3.412)	−0.187 (−0.782–0.409)	0.0597 (−0.567–0.686)
Number of observations	704	555	697	650

Notes: Adjusted models include all covariates listed in Table 1; dummy variables for each year of age are also included. Robust standard errors clustered at village level

**Table 4:** Malaria and educational outcomes, sickle cell trait only

	Child is currently in school (adjusted)	Grade attained conditional on age (adjusted)
>1 malaria episode	0.431** (0.212–0.877)	−0.256 (−0.701–0.190)
Number of observations	70	75

Notes: Adjusted models include all covariates listed in Table 1. Due to reduced sample size, adjusted regressions control for age and age squared rather than with dummy variables for each year of age. ** *p* < 0.05

**Fig. 1:**
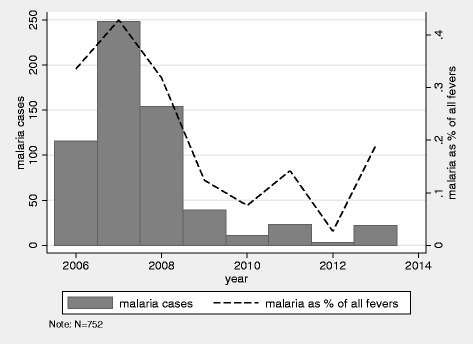
Number of malaria cases and percent of febrile illnesses confirmed as malaria by year

Figure 1 shows the number of malaria cases reported in each year. Highest incidence was observed for 2007 with a total of 248 cases; the number of cases declined rapidly after 2008, with less than 50 cases reported per year from 2009 to 2013. The percentage of febrile illnesses that were confirmed as malaria also declined sharply, from 34% in 2006 to 19% in 2013.

Table 2 shows the main results for fever and malaria incidence. SCT does not appear to have had a protective effect for episodes of fever. However, the presence of SCT was associated with reduction in the incidence of confirmed malaria cases by 29% in the fully adjusted models (Adjusted IRR 0.710, 95% CI [0.526, 0.959]).

The main results for educational outcomes are presented in Table 3. In the first two columns of Table 3 the odds ratios for current (as of May 2015) school attendance are shown. In columns 3 and 4, the difference in the highest grade attained conditional on students’ age in individuals with SCT compared to those without SCT is presented. There were no statistically significant associations for either of the two outcomes. The adjusted point estimates of the effect of SCT on highest grade attained was positive but not significant (0.0597), while adjusted odds ratio point estimate on the likelihood of being enrolled suggested a 42% greater likelihood of being in school among SCT individuals, but this was imprecisely estimated and not significant.

In Table 4, the sample is restricted to those with SCT, comparing educational attainment among those who have had at least one confirmed malaria episode versus those who did not have an episode of malaria over this period. In this subsample of 75 individuals, malaria was associated with reduced school enrollment (adjusted OR 0.431, 95% CI [0.212, 0.877]). The association between malaria and grade attainment in the SCT group was negative but not significant (adjusted grade difference −0.256, 95% CI [−0.701, 0.190].

## Discussion

The results presented in this paper yield three main results. First, similar to other studies, sickle cell trait (SCT) was found to be protective against malaria, with an estimated incidence reduction of 29% in fully adjusted models. Despite this reduced incidence of malaria, individuals with SCT did not show any greater educational attainment. However when analysis was restricted to individuals with SCT, exposure to malaria was associated with reduced school enrollment, even after adjusting for geographic and socioeconomic differences.

With respect to estimated effect sizes, the protective effect of SCT on malaria observed in this sample was notably smaller than recent estimates from Kenya, where a 50% reduction in the incidence of mild malaria, a 75% reduction in hospitalization and 90% reduction for severe malaria [3] was found, but similar to a recent study from Ghana, where a relative risk of 0.82 was found for subjects with SCT [5]. Interestingly, in both the Kenya study [3] and the study presented here, reduced exposure to confirmed cases of malaria did not result in reduced exposure to fevers more generally. One possible explanation for the relatively smaller protective effect observed in the study setting may have been the average age of subjects. An average age of 16 years old at the study endpoint implies that many participants were observed after the likely development of immunity to malaria for the entire 7 year period of febrile illness monitoring. The lower protective effect could also have been due to the rapidly dropping malaria burden, which has been observed in the study region after 2008 by several analyses [18, 20] and can very easily be seen in Fig. 1 of this paper as well. While the determinants of this decline are still not well understood, it seems likely that malaria control interventions such as Tanzania’s large scale bed net distribution campaign for all children under 5 years in 2008–2009 and for every sleeping space in 2010–2011, and the change in first line treatment to artemisinin combination therapy in 2007, played a major role. Other possible contributing factors include changes in climate, improved health service provision, and socio-economic development. The smaller estimated effects for fever incidence could be interpreted as evidence for SCT being associated with an increased incidence of other infections. However, the sample size of this study is not large enough to precisely estimate such differences.

Despite finding that SCT conferred protection from malaria, no associations between SCT and educational attainment were observed. This lack of association could partially have been due to the relatively small sample of 704 individuals, with only 81 SCT cases. (The sample was limited to 704 cases because these were the only individuals who were genotyped for SCD within the study area.) As a result, the study was only powered to reliably detect relatively large effect sizes. For example, in unadjusted models, the study was powered to detect an increase of 0.8 years of school attainment with 80% power. While the minimum detectable effect was smaller in adjusted models, because covariates such as age explain a great deal of the variation in schooling, power nonetheless remains a limitation of this study.

It is also possible that with half of the study population still in school, differences in educational attainment may not have fully emerged yet. Another possibility is that despite its frequent use in this literature [7–12], educational attainment is not an ideal measure for the underlying trait of cognitive improvement. While some studies have identified a link between malaria protection and schooling attainment [12], others have found cognition effects without educational attainment effects. For example a recent study which found links between birth year exposure to malaria eradication in Mexico and cognitive gains as measured by Raven progressive matrices nonetheless did not find schooling attainment gains [11]. In settings where poverty is a barrier to continued education, increased ability may not translate directly into increased educational attainment.

It is also worth highlighting that in the six villages studied, starting in 2007 malaria treatment was provided by community health workers, who used rapid diagnostic tests (RDTs) to diagnose malaria. The presence of trained CHWs may have reduced the risk of malaria cases progressing in severity, and thus lowered the overall impact of malaria exposure. This would have had the effect of dampening the sickle-cell trait-induced differences in malaria morbidity between HbAS (SCT) and HbAA groups. Another potential reason why increased education as a result of sickle cell trait was not observed could be because individuals with and without sickle cell trait differ on unobserved characteristics in addition to their differential susceptibility to malaria. Table 1 shows relatively modest differences across the two groups on observed characteristics, but other unmeasured or unobservable differences between HbAS (SCT) and HbAA households are possible. For example, individuals with sickle cell trait are more likely to have a sibling with sickle cell disease, a serious illness which could necessitate that family resources are devoted to medical care rather than education.

Finally, within the SCT group, malaria was associated with lower levels of school enrollment, even after controlling for a range of socioeconomic and demographic factors. This suggests that even in the SCT group, which was relatively protected from malaria, there may have been subpopulations which are particularly vulnerable to acute episodes of malaria that have deleterious effects on longer run social and developmental outcomes. This is an area that should be researched further.

Given the wide range of studies which suggest long run benefits to malaria protection in childhood [7–12], the relationship between SCT and long run cognitive development should be further investigated. This study points towards two potential avenues for future research. First, researchers could follow up on this or similar populations to determine whether SCT-induced protection from malaria translates into cognitive differences when measured directly via standard batteries of cognitive tests, rather than the proxy of educational attainment. Second, alternative empirical strategies can be applied to isolate the causal effect of reduced malaria morbidity on cognitive ability. In this study, just 145 out of 704 children were genotyped together with other members of their household, which was too small of a sample to estimate household fixed effects models. Future data collection efforts could be designed to exploit within-household variation on hemoglobin genotype, thereby eliminating the possibility that differences in household level characteristics such as wealth or parental education are confounding the hypothesized relationship of SCT to educational or cognitive outcomes.

## Conclusions

The results of this study suggest limited association between SCT and educational attainment. Given that SCT lowers incidence of malaria, this suggests that the causal impact of malaria morbidity on educational attainment, as measured by years in school and enrolment, was likely to have been relatively limited in this population. However, children who get malaria despite SCT also appeared to have lower levels of enrolment in education than their peers.

## Abbreviations

CORPs: Community-owned resource person
HbAA: Normal hemoglobin
HbAS: Heterozygous sickle hemoglobin
HbSS: Homozygous sickle hemoglobin
HDSS: Health and Demographic Surveillance System
PCD: Passive case detection
RBC: Red blood cell
RDTs: Rapid diagnostic tests
SCD: Sickle-cell disease
SCT: Sickle-cell trait
